# Comparing survival and clinical features of anti-MDA5 and anti-synthetase idiopathic inflammatory myopathy in a North American cohort

**DOI:** 10.3389/fmed.2026.1953868

**Published:** 2026-10-28

**Authors:** Ana Valle, Raisa Lomanto Silva, Monika Nukala, Mark M. Hammer, Tiffany Y.-T. Hsu, Misti Paudel, Paul F. Dellaripa

**Affiliations:** 1 Division of Rheumatology, Inflammation, and Immunity, Brigham and Women’s Hospital, Boston, MA, United States; 2 Harvard Medical School, Boston, MA, United States; 3 Department of Rheumatology, Allergy & Immunology, Massachusetts General Hospital, Boston, MA, United States; 4 Department of Radiology, Brigham and Women’s Hospital, Boston, MA, United States

**Keywords:** anti-MDA5, anti-synthetase syndrome, cohort, dermatomyositis, idiopathic inflammatory myopathies, mortality, myositis

## Abstract

**Introduction:**

Anti-synthetase syndrome (ASyS) and anti-MDA5 dermatomyositis (DM) cause significant morbidity and mortality due to interstitial lung disease (ILD). While large cohorts of these groups have been studied in Asia and Europe, data on clinical features and mortality risk within North America is limited. We aim to compare the mortality and clinical features between patients with anti-MDA5 DM and those with ASyS in a North American myositis cohort to identify predictors of mortality.

**Methods:**

A retrospective chart review was conducted in a large healthcare system. Patients were identified through specialty clinics, diagnosed with anti-MDA5 DM or ASyS by an expert clinician, and confirmed positive for anti-MDA5 or an ASyS antibody. Demographics, clinical features including baseline pulmonary function tests (PFTs) and high-resolution CT (HRCT), and treatment regimens were collected. Statistical analyses included t-test, Wilcoxon tests, Kaplan–Meier curves, and univariate Cox proportional hazards models.

**Results:**

One hundred patients (50% with anti-MDA5 DM, 50% ASyS) were analyzed. Over a median follow-up of 7.4 years, 18 patients died (8 in the anti-MDA5 group, 10 in the ASyS group). The 10-year survival rates of the two groups were similar. Age predicted mortality (*p* ≤ 0.001), but race, the presence of ulcerations, initial HRCT with >20% ILD, baseline percent predicted forced vital capacity <70%, or percent predicted diffusing capacity of the lungs for carbon monoxide <55% did not.

**Conclusion:**

Patients with anti-MDA5 DM and ASyS had similar survival rates. Older age at diagnosis predicted increased mortality, while baseline HRCT and PFT features did not. Further studies are needed to understand the variation in survival among patients with anti-MDA5 DM.

## Introduction

Idiopathic inflammatory myopathies (IIM, or myositis) are highly heterogeneous diseases that affect the muscles, skin, lungs, and heart, with unique immunological and demographic features (1). IIM-associated interstitial lung disease (ILD) has variable incidence depending on the myositis-specific autoantibody profile and disease phenotype, with cohorts of clinically amyopathic dermatomyositis (DM) reporting as high as 100% occurrence of ILD in these patients (2). ILD is responsible for the majority of hospitalizations, morbidity, and mortality in patients with myositis (3, 4). In addition, pulmonary symptoms may precede muscle weakness, posing a diagnostic challenge for clinicians (5).

In 2005, researchers from Japan were the first to describe an autoantibody recognizing a 140 kDa polypeptide associated with rapidly progressive (RP) ILD, later identified as an antibody against melanoma differentiation-associated protein 5 (anti-MDA5) (6). Since then, various cohorts from Asia and Europe have solidified the prognostic importance of this antibody and described the clinical characteristics and progression of patients with anti-MDA5 DM (7–11). A meta-analysis of published articles between 2000 and 2020 confirmed a higher prevalence of IIM-ILD in Asia compared with other continents (12). Detailed studies on the clinical characteristics of IIM-ILD, specifically anti-MDA5–positive ILD, in North America are rare. However, two single-center studies suggest that anti-MDA5 cases in North America have lower mortality when compared with Asian cohorts (13, 14).

Anti-synthetase syndrome (ASyS) is another subtype of IIM associated with ILD and marked by the presence of aminoacyl–tRNA synthetase antibodies. Anti-PL7 and anti-PL12 have been associated with RP–ILD and, at times, also present with isolated ILD at presentation (15, 16).

Direct comparisons of anti-MDA5 DM and ASyS are few, despite their potential phenotypic overlap. The available literature stems from large cohorts in Asia and Europe (8, 17). We also lack an understanding of the clinical features and outcomes of patients with anti-MDA5 DM in North America, which previous studies suggest may differ from other parts of the world. To characterize the phenotypes associated with IIM-ILD–associated antibodies, we compared the clinical features and mortality risk between patients with anti-MDA5 DM and those with ASyS in a North American myositis cohort, with the goal of identifying predictors of mortality. We hypothesized that individuals with anti-MDA5 DM would have a higher mortality rate compared to individuals with ASyS.

## Materials and methods

### Setting and participants

We conducted a retrospective chart review using electronic health records (EHRs) from the Mass General Brigham (MGB) healthcare system from 1 January 2000 through 6 September 2024. Patients were identified through the pulmonary and rheumatology clinics. Inclusion criteria were a diagnosis of anti-MDA5 DM or ASyS by an MGB clinical expert and seropositivity for anti-MDA5 or an ASyS antibody (Jo-1, PL-7, PL-12, EJ, OJ). Anti-MDA5 testing at Brigham and Women’s Hospital was performed using the Oklahoma Medical Research Foundation (OMRF) Comprehensive Myositis Autoantibody Profile from 2005 to 2023. The OMRF uses immunoprecipitation and Western blotting for its assays (18). Since 2023, an in-house immunoblot assay has been used. Additional commercial assays utilized included the MyoMarker 3 or MyoMarker 3 Plus Profile (RDL Reference Laboratory) and the EUROLINE Autoimmune Inflammatory Myopathies 16 Ag immunoassay (Euroimmun) (19). Chart review was carried out between January 2026 and May 2026.

Our primary outcome was mortality, specifically, time from IIM diagnosis to death. Age, race, the presence of ulcerative lesions, baseline high-resolution CT (HRCT) findings, and pulmonary function test (PFT) features were evaluated as predictors of mortality. The initial HRCT finding of interest was ILD involving >20% of the lung parenchyma based on the work by Goh et al. (20). The PFT findings of interest were an initial percent predicted forced vital capacity (FVC) of <70% and diffusing capacity of the lungs for carbon monoxide (DLCO) <55%, as these features have been utilized for prognostication in systemic sclerosis-associated ILD (21, 22).

### Clinical data

Baseline demographics (age, sex, race, and ethnicity), disease characteristics (symptom onset, diagnosis date, disease duration), serologic profile, clinical manifestations by organ system, and treatment data were extracted from our EHR system. Symptom onset was defined as the first occurrence of dyspnea, rashes, proximal weakness, or inflammatory arthritis in the months or years preceding the IIM diagnosis without another explanation. The diagnosis date was determined based on the first clinical note in which a clinical expert (e.g., pulmonologist, rheumatologist, dermatologist, or neurologist) stated anti-MDA5 DM or ASyS was the most likely diagnosis. In addition to ASyS and anti-MDA5 antibodies, the presence of antibodies against TIF-1*γ*, NXP2, Mi-2, SAE1, SRP, Ku, U1 snRNP, U2 snRNP, U3 snRNP, SSA/Ro, SSB/La, Ro-52, Ro-60, PM/Scl-100, and Scl-70 was collected. All antibody serologies were recorded as present, absent, or unknown. Muscular clinical manifestations were determined by the presence of weakness on manual muscle testing at any point during the patient’s disease course and peak creatine phosphokinase (CPK) value. Cutaneous manifestations documented included calcinosis, Gottron papules and sign, the heliotrope rash, the holster sign, inverse Gottron papules, mechanic's hands, the malar rash, poikiloderma, the shawl sign, the V sign, periungual erythema, ulcerative skin lesions, oral and nasal ulcerations, and other unspecified rashes. The presence of Raynaud phenomenon and secondary ulceration and digital loss due to ischemia was also recorded. Gastrointestinal involvement was defined as dysphagia endorsed by the patient. A history of myocarditis and/or pericarditis was determined based on documented clinical suspicion leading to treatment or cardiac magnetic resonance imaging. The presence of pulmonary hypertension was determined by echocardiogram or right heart catheterization.

Pulmonary involvement was assessed by 1) baseline PFTs, 2) baseline HRCT findings, 3) use and amount of supplemental oxygen, 4) advanced airway (e.g., high-flow oxygen, mechanical ventilation), and 5) transplant evaluation. Baseline PFTs were defined as the earliest PFTs available in the medical record, selected to capture predisease lung status and identify unique functional features rather than measurements already influenced by advanced ILD in cases of diagnostic delay. HRCT findings were evaluated by two fellowship-trained thoracic radiologists, who initially evaluated the CTs independently, followed by resolution of discrepancies by consensus. In addition, smoking status as former, current, or never smoker was recorded. Packs per year were determined when possible. The number of steroid pulses (defined as IV methylprednisolone >500 mg daily for two days or more) administered was recorded. Disease-modifying antirheumatic drugs, advanced therapeutics (including biologics, JAK inhibitors, etc.), and antifibrotic agents were recorded based on previous or current use. Missing data were assumed not to be present.

### Statistical analysis

Means (standard deviations), numbers (percentages), and medians (interquartile range, IQR) were calculated to describe demographics, serologic profiles, disease characteristics, and treatment data. All descriptive statistics were reported for the total cohort and the distribution of features between patients who were anti-MDA5–positive and those with ASyS. Differences in clinical manifestations were compared using chi-square tests or Fisher's exact tests and *t*-tests or Wilcoxon rank-sum tests for categorical and continuous variables, respectively. Due to the number of comparisons, demographics were adjusted using the Holm–Bonferroni method, while organ involvement was adjusted using the false discovery rate method. The Kaplan–Meier method was used to create survival curves. The survival curves were truncated at year 10 based on the limited number of patients remaining at risk after this time. We determined median survival time and survival probabilities between anti-MDA5 DM and ASyS at years 1, 5, and 10. If statistical differences between the anti-MDA5 and ASyS groups were identified, we would limit identification of predictors of mortality to the group with the highest mortality. In the absence of a meaningful between-group difference, pooling of the groups was utilized to preserve statistical power and yield a more stable estimate of overall risk. Univariate Cox models were utilized to identify predictors of mortality. The risk factors of age, race, the presence of ulcerative lesions, baseline HRCT findings, and PFT features were analyzed as predictors of mortality. Hazard ratios and *p*-values were calculated for each predictor. Based on 18 events within the sample of 100 patients, the maximum number of predictors our model could accommodate was two. All statistical analyses were performed using R (23). All *p*-values ≤0.05 were considered significant.

### IRB approval

This study was approved and deemed exempt by the Mass General Brigham Internal Review Board, Protocol #2023P003602.

## Results

A total of 100 patients were identified and analyzed. Half of these patients were positive for anti-MDA5 antibodies, while the other half were positive for anti-synthetase antibodies. Overall, the mean (SD) age was 56.9 (14.9) years, with the majority of patients being female (70%), White (65%), and of non-Latinx (83%) ethnicity (Table 1). Those with anti-MDA5 disease were more likely to be of Hispanic ethnicity in comparison with those with ASyS (7 vs. 0, *p* = 0.018). Sex and race distribution were similar between the two groups. Those with ASyS had a longer median (IQR) time from symptom onset to diagnosis [anti-MDA5 with 121 (60–183) days vs. ASyS with 153 (68–518) days, *p* = 0.05] (Table 1).

**Table 1:** Demographics based on anti-MDA5 and anti-synthetase antibody status and of total cohort.

Demographics		Anti-MDA5 (*N* = 50)	ASyS (*N* = 50)	Total (*N* = 100)	*P* - Value
Age, mean (SD), years		56.7 (15.8)	60.2 (16.7)	56.9 (14.9)	0.689
Sex, *n* (%)	Female	36 (70)	34 (70)	70 (70)	0.827
	Male	14 (30)	16 (30)	30 (30)	
Race, *n* (%)	Asian	6 (12)	4 (8)	10 (10)	0.641
	Black	5 (10)	13 (26)	18 (18)	
	White	35 (70)	30 (60)	65 (65)	
	Other	4 (8)	3 (6)	7 (7)	
Latinx ethnicity, *n* (%)	Yes	7 (14)	0 (0)	7 (7)	**0** **.** **018**
	No	38 (76)	45 (90)	83 (83)	
	Unknown	5 (10)	5 (10)	10 (10)	
Time to diagnosis, median (IQR), days		121 (60–183)	153 (68–518)	122 (61–305)	**0.050**
Disease duration, median (IQR), years		6.5 (2.4–10.4)	8.3 (4.3–14.8)	7.4 (4.1–11.5)	**0.026**
Antibody profile, *n* (%)	MDA5	50 (100)	0 (0)	50 (50)	
	Jo-1	0 (0)	30 (60)	30 (30)	
	OJ	0 (0)	3 (6)	3 (3)	
	EJ	0 (0)	1 (2)	1 (1)	
	PL-7	0 (0)	9 (18)	9 (9)	
	PL-12	0 (0)	9 (18)	9 (9)	
	TIF-1 gamma	0 (0)	0 (0)	0 (0)	
	NXP2	1 (2)	0 (0)	1 (1)	
	Mi 2	1 (2)	1 (2)	2 (2)	
	SAE1	0 (0)	0 (0)	0 (0)	
	SRP	1 (2)	0 (0)	1 (1)	
	PM-Scl 100	0 (0)	0 (0)	0 (0)	
	Scl 70	0 (0)	0 (0)	0 (0)	
	Ku	2 (4)	0 (0)	2 (2)	
	U1 snRNP	1 (2)	0 (0)	1 (1)	
	U2 snRNP	0 (0)	0 (0)	0 (0)	
	U3 snRNP	0 (0)	0 (0)	0 (0)	
	Ro	7 (14)	7 (14)	14 (14)	
	La	0 (0)	2 (4)	2 (2)	
	Ro 52	10 (20)	15 (30)	25 (25)	
	Ro 60	3 (6)	2 (4)	5 (5)	

Bolded *p*-values signifiy statistical significance.

Patients with anti-MDA5 DM had significantly lower median (IQR) peak CPK in comparison to individuals with ASyS: 146 (67–418) U/L vs. 650 (230–2411) U/L (*p* < 0.0001). This lower peak CPK level corresponded with less clinical weakness based on manual muscle testing, although this was not statistically significant (Table 2). Typical DM rashes (Gottron's papules, Gottron's sign, and V signs) occurred in individuals with anti-MDA5 more frequently. Inverse Gottron's papules, ulcerative skin lesions, and other ulcerative lesions (whether from skin, mucosa, or Raynaud's phenomenon) were also more likely in patients with anti-MDA5 (Table 2). The number of deaths between the two groups was similar (*p* = 1.00). However, causes of death varied; individuals with ASyS expired from cancer, ILD, infection, cardiac, or dementia, while 100% of anti-MDA5 DM patients who died did so due to their ILD (*p* = 0.02, Table 2).

**Table 2:** Clinical manifestations of individuals based on anti-MDA5 and anti-synthetase status (excluding pulmonary characteristics and manifestations).

Clinical manifestations		Anti-MDA5 (*N* = 50)	ASyS (*N* = 50)	*P*-value
Muscle manifestations	Clinical weakness, N (%)	15 (30)	27 (54)	0.058
	Peak CPK value, median (IQR) in units per liter (U/L)	150 (67–458)	635 (228–2,299)	**<0** **.** **0001**
Cutaneous manifestations, *n* (%)	Calcinosis	5 (10)	1 (2)	0.234
	Gottron's papules	42 (84)	14 (28)	**<0**.**0001**
	Gottron's sign	28 (56)	2 (4)	**<0**.**00001**
	Heliotrope	16 (32)	9 (18)	0.203
	Holster	7 (14)	2 (4)	0.203
	Inverse Gottron's papules	22 (44)	0 (0)	**<0**.**0001**
	Malar rash	19 (38)	6 (12)	**0.009**
	Mechanic's hands	21 (42)	27 (54)	0.338
	Poikiloderma	7 (14)	1 (2)	0.095
	Shawl sign	16 (32)	4 (8)	**0.009**
	V sign	26 (54)	4 (8)	**<0**.**0001**
	Periungual erythema	10 (20)	10 (20)	1.00
	Oral ulcers	17 (34)	0 (0)	**0.001**
	Ulcerative skin lesions	24 (48)	0 (0)	**<0**.**0001**
	Raynaud's phenomenon	24 (48)	32 (64)	0.203
	Ulceration	7/24 (29)	4/32 (13)	
	Digit loss	3/24 (13)	1/32 (3)	
	Any ulcerative disease^a^	30 (60)	4 (8)	**<0**.**0001**
GI manifestations, *n* (%)	Dysphagia	16 (32)	22 (44)	0.606
Cardiac manifestations, *n* (%)	Myocarditis	0 (0)	6 (12)	0.080
	Pericarditis	3 (6)	5 (10)	0.715
Deaths, *n* (%)		8 (16)	10 (20)	1.00
Causes of death, *n* (%)	Respiratory failure from ILD	8/8 (100)	3/10 (30)	**0**.**020**
	Infection	0 (0)	2/10 (20)	
	Cardiac (VF, PEA, MI, etc.)	0 (0)	3/10 (30)	
	Dementia	0 (0)	1/10 (10)	
	Cancer	0 (0)	1/10 (10)	

GI, gastrointestinal; VF, ventricular fibrillation; PEA, pulses electrical activity; MI, myocardial infarction.

Bolded *p*-values signifiy statistical significance.

a Any ulcerative disease includes skin, oral, nasal ulcers and ulceration secondary to Raynaud's phenomenon.

Presenting PFT findings were generally similar (Table 3). However, patients with anti-MDA5 DM who died had a baseline percent predicted DLCO of 48%, while those who survived had a baseline percent predicted DLCO of 62%. Interestingly, patients with ASyS often had their initial PFTs completed 1 year prior to diagnosis, whereas those with anti-MDA5 DM had them completed approximately 3.5 years after diagnosis (Table 3). Significant differences in HRCT findings were noted, with a higher frequency of diffuse alveolar damage (DAD) and a lower prevalence of non-specific interstitial pneumonia (NSIP) in patients with anti-MDA5 DM in comparison to those with ASyS (*p*-values = 0.004, 0.002, respectively).. The proportion of patients with a presenting HRCT scan showing with > 20% ILD was similar between the two groups (*p* = 0.139) (Table 3). None of the individuals with ASyS required advanced airway support, while 6 (12%) patients with anti-MDA5 DM did; this trend was not significant (*p* = 0.114). Treatment regimens between the two groups were generally similar, with a high utilization of antirheumatic drugs and biologics and infrequent use of antifibrotic agents. However, pulse doses of corticosteroids and intravenous immunoglobulin were used more frequently in patients with anti-MDA5 DM (Table 4).

**Table 3:** Pulmonary characteristics and manifestations of individuals based on anti-MDA5 and anti-synthetase status.

Pulmonary manifestations		Anti-MDA5 (*N* = 50)	ASyS (*N* = 50)	*P*-value
Pulmonary hypertension, *n* (%)		8 (16)	13 (26)	0.554
Initial PFTs, mean (SD)	FEV1 (mL)	2.5 (1.06)	2.0 (0.65)	0.129
	FEV1 (% predicted)	83 (22.3)	74 (17.4)	0.114
	FVC (mL)	3.0 (1.25)	2.6 (0.81)	0.127
	FVC (% predicted)	80.3 (23.1)	70.3 (19.8)	0.127
	DLCO (L)	14.7 (23.7)	14.8 (6.41)	0.730
	DLCO (% predicted)	58 (24.0)	61 (20.6)	0.707
Initial HRCT findings, *n* (%)	DAD	10 (20)	0 (0)	**0**.**004**
	NSIP	12 (24)	32 (64)	**0**.**002**
	UIP	4 (8)	2 (4)	0.203
	OP	19 (38)	15 (30)	0.609
	ILD > 20%	21 (42)	22 (44)	1.00
	Fibrosis	12 (24)	22 (44)	0.139
Time from diagnosis to PFTs, median (IQR), days		1,192 (−498 to 2,324)	−561 (−2,859 to –1,804)	**0**.**010**
Supplemental oxygen, *n* (%)		13 (26)	16 (32)	0.637
Average L/min		4.1	4.4	
Advanced airway support, *n* (%)		6 (12)	0 (0)	0.114
Transplant evaluation, *n* (%)		12 (24)	8 (16)	0.637
Previous smoker, *n* (%)		15 (30)	18 (36)	0.637
Average pack per year history		17.0	15.9	

FEV1, forced expiratory volume in 1 s; DAD, diffuse alveolar damage; NSIP, non-specific interstitial pneumonia; UIP, usual interstitial pneumonia; OP, organizing pneumonia.

Bolded *p*-values signifiy statistical significance.

**Table 4:** Comparison of current and past treatment among individuals with anti-MDA5 DM and ASyS.

Treatment		Anti-MDA5 (*N* = 50)	ASyS (*N* = 50)	Total (*N* = 100)
Hydroxychloroquine, *n* (%)	Current use	7 (14)	5 (10)	12 (12)
	Past use	9 (18)	8 (16)	17 (17)
	Never used	34 (68)	37 (74)	71 (71)
Mycophenolate mofetil and its analogs, *n* (%)	Current use	24 (48)	21 (42)	45 (45)
	Past use	12 (24)	16 (32)	28 (28)
	Never used	14 (28)	13 (26)	27 (27)
Methotrexate, *n* (%)	Current use	1 (2)	4 (8)	5 (5)
	Past use	17 (34)	16 (32)	33 (33)
	Never used	32 (64)	30 (60)	62 (62)
Leflunomide, *n* (%)	Current use	0 (0)	0 (0)	0 (0)
	Past use	1 (2)	2 (4)	3 (3)
	Never used	49 (98)	48 (96)	97 (97)
Azathioprine, *n* (%)	Current use	0 (0)	3 (3)	3 (3)
	Past use	10 (20)	18 (36)	28 (28)
	Never used	40 (90)	29 (58)	69 (69)
Tacrolimus, *n* (%)	Current use	7 (14)	1 (2)	8 (8)
	Past use	5 (10)	5 (10)	10 (10)
	Never used	38 (76)	44 (88)	82 (82)
Rituximab, *n* (%)	Current use	13 (26)	6 (12)	19 (19)
	Past use	12 (24)	20 (40)	32 (32)
	Never used	25 (50)	24 (48)	82 (82)
Cyclosporine, *n* (%)	Current use	3 (6)	1 (2)	4 (4)
	Past use	6 (12)	5 (10)	11 (11)
	Never used	41 (82)	44 (88)	85 (85)
Intravenous immunoglobulin, *n* (%)	Current use	20 (40)	3 (6)	23 (23)
	Past use	13 (26)	12 (24)	25 (25)
	Never used	17 (34)	35 (70)	52 (52)
JAK inhibitors, *n* (%)	Current use	8 (16)	0 (0)	8 (8)
	Past use	3 (6)	1 (2)	4 (4)
	Never used	39 (78)	49 (98)	88 (88)
Abatacept, *n* (%)	Current use	0 (0)	0 (0)	0 (0)
	Past use	1 (2)	0 (0)	1 (1)
	Never used	49 (98)	50 (100)	99 (99)
Nintedanib, *n* (%)	Current use	1 (2)	3 (6)	4 (4)
	Past use	3 (6)	5 (10)	8 (8)
	Never used	46 (94)	42 (84)	88 (88)
Pirfenidone, *n* (%)	Current use	0 (0)	0 (0)	0 (0)
	Past use	0 (0)	2 (4)	2 (2)
	Never used	0 (0)	48 (96)	98 (98)
Nerandomilast, *n* (%)	Current use	0 (0)	0 (0)	0 (0)
	Past use	0 (0)	0 (0)	0 (0)
	Never used	0 (0)	0 (0)	0 (0)
Pulse steroids (1 g × 3 days), *n* (%)		13 (26)	7 (14)	20 (20)

Of the 100 patients, 90% survived 1 year after diagnosis, 88% survived 5 years after diagnosis, and 86% survived 10 years after diagnosis (Supplementary Figure 1). At years 1, 5, and 10, survival rates were 88%, 86%, and 86% for the anti-MDA5 DM group, versus 94%, 90%, and 86% for the ASyS group. Comparison of these two groups at years 1, 5, and 10 did not reveal a difference in survival at these time points (Figure 1). Among the entire cohort, a univariate Cox proportional hazards analysis found age to be a predictor of mortality. With each 1-year increase in age at the time of diagnosis, there is an increase in mortality by a hazard ratio of 1.08 (CI 1.03–1.12, *p* = 0.0009). Race, the presence of any ulcerative lesions (HR 0.30, 95% CI 0.07–1.32, *p* = 0.112), presenting HRCT with >20% ILD, baseline percent predicted FVC of <70%, and DLCO <55% were not found to be predictors of mortality in this cohort (Supplementary Table 1).

**Figure 1:**
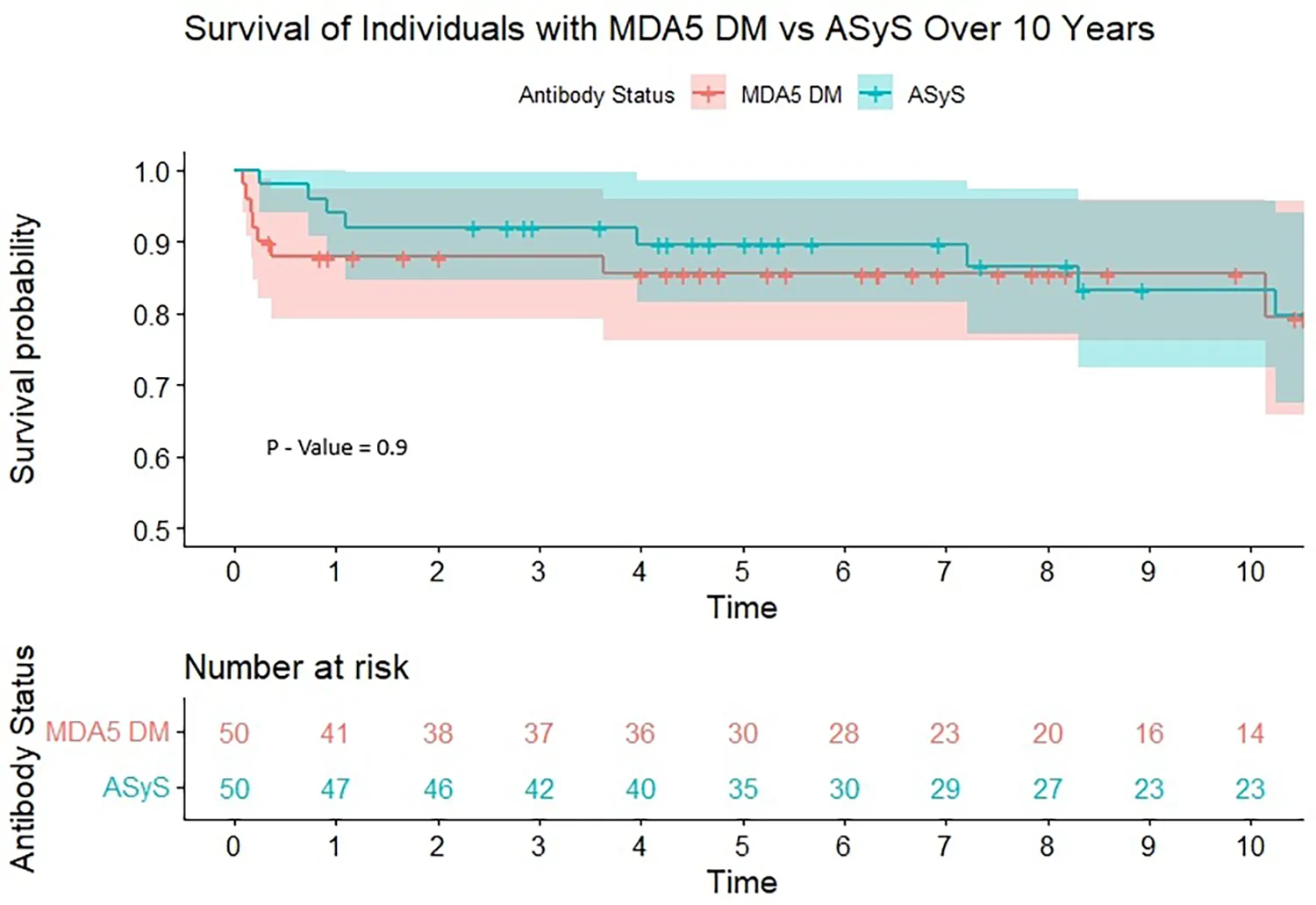
Comparison of survival of individuals with anti-MDA5 DM and ASyS at years 1, 5, and 10 after diagnosis.

## Discussion

In a US-based cohort, we found the mortality of patients with anti-MDA5 DM and those with ASyS to be comparable during the first 10 years after diagnosis, with an overall 10-year survival rate of 86% for the entire cohort. Age was a predictor of mortality, while race, ulcerative lesions, >20% ILD on initial HRCT, and baseline percent predicted FVC <70% and DLCO <55% were not. Despite similar survival rates, patients with anti-MDA5 DM still exhibited signs of advanced lung disease based on their need for advanced airway support and the fact that all deaths in this group were secondary to ILD. Although specific race or pulmonary features did not predict mortality, the frequencies of classic DM lesions, ulcerative skin lesions, and any ulceration (of the mouth, nose, skin, or secondary to Raynaud phenomenon) were significantly different between patients with anti-MDA5 DM and those with ASyS. We also found significant differences in muscle involvement.

Patients with anti-MDA5 DM had a first-year mortality rate of 12%. This differs from multiple previous studies that report anti-MDA5 DM mortality rate of 50%–80% within the first year of diagnosis (9, 24, 25). These data were based on cohorts in Asia, where patients typically have an aggressive ILD phenotype and require triple therapy (8, 9, 25). However, a French anti-MDA5 cohort reported three distinct phenotypes based on RP–ILD, inflammatory arthritis, and vasculopathy features (26). Those with RP–ILD had a much higher mortality rate in comparison with the other two phenotypes (24). This phenotypic variation and its association with mortality were echoed by a Canadian study of predominantly Asian patients (27). It is plausible that our cohort largely consisted of the latter two phenotypes with less severe ILD, given that we identified individuals who survived to attend outpatient visits. However, our anti-MDA5 DM cohort had a higher frequency of advanced airway support, and all deaths were secondary to respiratory failure from ILD, suggesting that we did capture a subset with RP–ILD. This is further supported by a lower percent predicted DLCO in patients with anti-MDA5 DM who died compared with those who survived.

The similarity in survival rates between patients with anti-MDA5 DM and those with ASyS reported here differs from that reported in some previous studies. In particular, Liu et al. directly compared these two IIM subtypes in their Taiwanese cohort and found that patients with anti-MDA5 antibodies had a higher mortality rate of 1.95 per person-year than those with Jo-1 antibodies (0.094/person-year) (8). Our findings align more with the experiences of other institutions within our geographic region. Cheah et al.'s California-based cohort of patients with anti-MDA5 DM ILD also had a low mortality rate of 2/24 (8.3%) over a mean follow-up period of 4 years (14). Tiniakou et al. at the Johns Hopkins Myositis Center reported a low mortality rate of 5/52 (9.6%) in patients with anti-MDA5 DM. When compared with anti-Jo-1–positive patients, the frequency of all-cause mortality was similar (7/51, 14%) (13). We hypothesize that the lower mortality within the United States and other North American cohorts is due to a smaller subset of patients with anti-MDA5 DM having RP–ILD compared with Asian cohorts. While we did not formally assess the percentage of patients with anti-MDA5 DM who met criteria for rapidly progressive ILD, this is an important next step to evaluate our hypothesis (28).

Our patients with anti-MDA5 DM exhibited clinical features similar to those described by Tiniakou et al.: a lower maximum CPK level and less clinical muscle weakness but more frequent need for mechanical ventilation compared with those with ASyS and a high frequency of classic DM rashes (13). These clinical features align with the expected phenotype of anti-MDA5 DM. We also note that baseline DAD on HRCT was only seen in our cohort in patients with anti-MDA5 DM. In previous studies, DAD has been noted as a harbinger of poor prognosis (29–31). However, studies have been limited by small sample sizes, and in multivariable analyses, they have shown mixed results in terms of predicting poor outcomes (29, 31). Altogether, these clinical similarities suggest our patients with anti-MDA5 DM share phenotypes with other reported cohorts. While we did not evaluate the effect of therapies on mortality or pulmonary outcomes in this cohort, we acknowledge that evolving and emerging therapies may affect mortality compared with older studies that employed less targeted or less aggressive therapies. This is a crucial area that requires further exploration as we strive to understand the variables that impact survival in anti-MDA5 DM.

This study has several limitations. It is a single-center study with a small sample size and rare events, which limited the number of clinical manifestation comparisons we could carry out. It is possible that a larger sample size would be adequately powered to detect differences in mortality between the two groups. As a retrospective chart review, this study was limited by the availability of PFTs and HRCT scans at the time of diagnosis, which may account for the differences in the time between the diagnosis date and timing of initial PFTs between the two groups. We did not evaluate how many patients met criteria for rapidly progressive ILD, although the frequency of these lung findings likely influenced mortality. There is also an inherent selection bias, as patients who were seen in the ambulatory setting likely had stable disease, whereas those with more rapid ILD were less likely to survive to outpatient follow-up. In addition, patients were identified over a broad time span (2000 to 2024), during which anti-MDA5 antibody testing methods varied.

In summary, this study provides a direct comparison of clinical features and mortality between patients with anti-MDA5 DM and those with ASyS in North America. Our findings suggest that the clinical trajectory of anti-MDA5 DM cases in North America may differ from that observed in other geographic regions due to a decreased frequency of RP–ILD. We found that patients with anti-MDA5 DM and those with ASyS had similar survival rates, which worsened with older age at diagnosis. Further studies are warranted to clarify the trajectory and variables that impact survival in anti-MDA5 DM, particularly regarding the importance of diffuse alveolar damage we identified.

## Data Availability

The raw data supporting the conclusions of this article will be made available by the authors, without undue reservation.
