# Supplementary material for: Comparing survival and clinical features of anti-MDA5 and anti-synthetase idiopathic inflammatory myopathy in a North American cohort
Source: Front Med (Lausanne). 2026 Oct 28;13:1953868. doi: 10.3389/fmed.2026.1953868 (PMC13751478; doi:10.3389/fmed.2026.1953868)
Supplement: Supplementary file 1 [file Datasheet1.docx]

**Supplementary Material**

Figure 1. The survival rate of the entire cohort at year 1, year 5, and year 10 following diagnosis.

Supplemental Table 1. Hazard ratios (HR) and p-values of predictors of mortality.

| Variable | HR (95% CI) | P-Value |
| --- | --- | --- |
| Age | 1.08 (1.03-1.12) | <0.001 |
| Race | 1.49 (0.94-2.38) | 0.092 |
| Any ulcerative lesions | 0.30 (0.07-1.32) | 0.112 |
| Presenting HRCT with > 20% ILD | 2.07 (0.81-5.26 | 0.130 |
| Baseline percent predicted FVC of < 70% | 1.40 (0.51-3.87) | 0.517 |
| Baseline DLCO < 55% | 1.46 (0.69-0.42) | 0.551 |
| Where HR = hazard ratio, CI = confidence interval | | |
